# Network Desynchronization with Sine Waves: from Synchrony to Asynchrony by Periodic Stimulation

**DOI:** 10.1002/advs.202414602

**Published:** 2025-06-18

**Authors:** Joana Covelo, Martina Cortada, Gianni V. Vinci, Maurizio Mattia, Maria V. Sanchez‐Vives

**Affiliations:** ^1^ Institut d'Investigacions Biomèdiques August Pi i Sunyer (IDIBAPS) Barcelona 08036 Spain; ^2^ Facultat de Física Universitat de Barcelona (UB) Barcelona 08028 Spain; ^3^ Natl. Center for Radiation Protection and Computational Physics Istituto Superiore di Sanità Rome 00161 Italy; ^4^ ICREA Barcelona 08193 Spain; ^5^ Facultat de Medicina i Ciències de la Salut Universitat de Barcelona (UB) Barcelona 08036 Spain

**Keywords:** cortical circuits, electric fields, network dynamics, neuromodulation, slow waves, tACS, tDCS

## Abstract

Understanding how brain stimulation interacts with the brain's internal dynamics is crucial for developing effective neuromodulation protocols. Here we explore the effects of exogenous alternating current (AC) fields across various amplitudes and frequencies on cortical slices expressing spontaneous slow oscillations. Cortical network entrainment occurs within an Arnold tongue‐like region centered at the endogenous frequency. However, slightly detuned periodic stimulation of higher frequency leads to a desynchronized regime, revealing a novel approach for disrupting pathological synchronicity. The introduction of an additional direct current (DC) offset expands the modulatory ranges, facilitating the achievement of either entrainment or desynchronization, depending on the DC offset's polarity. The experimental observations are quantitatively reproduced by a computational model of spiking neurons, suggesting that the interaction between nonlinear oscillators can predict the network's response to AC fields. Besides an improved understanding of cortical dynamics and its interaction with exogenous electric fields, a robust protocol with potential clinical applications in pathological conditions is presented.

## Graphical abstract

Exogenous alternating current fields are explored and interact with cerebral cortex slow waves in vitro and in silico. Varying frequency and amplitude of sine waves result in distinct network dynamics, from phase‐locking and entrainment to desynchronization. These responses occupy specific regions within the parameter space. A more robust modulatory protocol is achieved by adding a direct current offset. These findings open new avenues for neuromodulation.

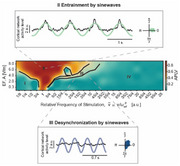

## 1 Introduction

Brain oscillations often coexist concurrently at different frequencies, reflecting synchronized processes that orchestrate the way information is processed and transmitted within neuronal cortical networks in a state‐dependent manner.^[^ ^1^, ^2^ ^]^ The cerebral cortex exhibits self‐sustained oscillatory activity even in the absence of external stimuli, a phenomenon governed by fundamental principles of nonlinear dynamics.^[^ ^3^, ^4^, ^5^ ^]^ Oscillatory behavior is a universal phenomenon observed across a wide range of systems, including physical, chemical, neural, and social domains.^[^ ^6^, ^7^, ^8^, ^9^, ^10^, ^11^ ^]^ Despite differences in scale and underlying mechanisms, many of these systems share common principles, offering insightful parallels that enhance our understanding of neural dynamics.

In the case of the brain, the states of slow‐wave sleep (SWS) and deep anesthesia are dominated by slow oscillations (SO), where the synchronous activity of large neuronal populations alternates between active (Up states) and silent (Down states) periods at a frequency of <1 Hz.^[^ ^12^, ^13^ ^]^ Research suggests that SWS is crucial for numerous physiological processes, including brain plasticity, metabolism, and immune system functioning.^[^ ^14^, ^15^, ^16^ ^]^ SWS deterioration increases with age and has been associated with multiple neuropsychiatric disorders, such as dementia, schizophrenia, insomnia, and major depression.^[^ ^17^ ^]^ Pathological SO activity has also been recorded in perilesional areas of acute ischemic cortical stroke patients^[^ ^18^, ^19^ ^]^ traumatic brain injury^[^ ^20^, ^21^, ^22^ ^]^ and in unresponsive wakefulness syndrome patients.^[^ ^23^ ^]^ Hence, neuromodulation targeting SO may be valuable not only for the understanding of basic physiology but also for the development of potential clinical applications.^[^ ^24^ ^]^

Transcranial alternating current stimulation (tACS) is a non‐invasive neuromodulation technique that usually aims at targeting neural oscillations.^[^ ^25^ ^]^ tACS consists of the delivery of low‐intensity electric currents to the scalp, generating a rhythmically fluctuating electric field (EF).^[^ ^26^, ^27^ ^]^ These weak EFs can lead to periodic sub‐threshold membrane depolarization, which is amplified by a dynamic network activity.^[^ ^2^, ^28^ ^]^ As such, the effects of tACS on brain dynamics will depend on the level of activity of neuronal populations that generate the network oscillations and are therefore frequency‐specific.^[^ ^2^ ^]^

Computational^[^ ^29^, ^30^, ^31^ ^]^ and experimental^[^ ^32^ ^]^ data suggest that the tACS stimulation frequency most effective at entraining neural oscillations is the frequency of the endogenous neural oscillation. With an increase in stimulation amplitude, the range of frequencies leading to entrainment widens, forming a triangular region of high synchrony between the exogenous AC field and the endogenous oscillations; that is, an Arnold tongue.^[^ ^32^ ^]^ In particular for SO, several studies have reported maximum entrainment when stimulating with alternating current (AC) fields that match the endogenous frequency of the target SO,^[^ ^29^, ^33^, ^34^ ^]^ which has been shown to improve memory consolidation.^[^ ^35^ ^]^ When considering other frequencies, tACS application is associated with improvement in the performance of working‐memory tasks in elderly people by enhancing theta synchronization^[^ ^36^ ^]^ and with the enhancement of multitasking performance in healthy adults associated with an enhancement of frontal theta, alpha, and beta oscillations.^[^ ^37^ ^]^ Additionally, in schizophrenia, which is associated with reduced alpha oscillations, tACS‐induced enhancement of alpha oscillations is associated with clinical improvement of auditory hallucinations.^[^ ^38^ ^]^

However, enhancing endogenous oscillations might not always be beneficial. In brain disorders characterized by hypersynchrony, such as depression, epilepsy, and Parkinson's disease,^[^ ^39^, ^40^, ^41^ ^]^ brain function could potentially be restored by attenuating or resetting the pathological oscillations,^[^ ^26^, ^42^ ^]^ for example, in brain injury.^[^ ^43^ ^]^ For these reasons, it is crucial to understand the precise mechanisms through which weak alternating currents interact with endogenous network activity patterns and determine the optimal parameters of stimulation for the desired noninvasive electrotherapeutic strategy.

Our objective was to investigate how exogenous brain stimulation interacts with the brain's internal dynamics. This is important to better understand network rhythmic activity, as well as the linear and nonlinear interactions between these different exogenous and endogenous oscillators. To this end, we explored the interaction between sinusoidal electric fields and cortical SO in a wide range of stimulation parameters. We applied external AC fields with various amplitudes and frequencies to brain slices exhibiting spontaneous SO, enabling a detailed investigation of the resulting complex cortical dynamics. Entrainment was achieved within a range of the stimulation parameters, near the endogenous value of SO frequency. However, stimulating parameters slightly above the intrinsic oscillation induced network desynchronization.

Recognizing the clinical value of desynchronization and based on our results, we propose a robust protocol that incorporates a direct current (DC) offset in addition to the AC stimulation. Weak DC fields induce an orientation‐dependent subthreshold shift in the somatic transmembrane voltage either toward depolarization (anodal) or hyperpolarization (cathodal), therefore affecting the probability of action potential firing.^[^ ^33^, ^44^ ^]^ This combined approach aims to enhance the induction of desynchronized states more robustly, offering a potentially more effective method for modulating cortical dynamics. This protocol not only improves the efficacy of inducing desynchronization but also enhances entrainment, depending on whether the DC offset is negative or positive, respectively. This dual capability increases its therapeutic versatility, making it adaptable to different neurological conditions. The richness of the tissue response to this combination of stimuli is quantitatively captured by a network model of spiking neurons. This model enables us to analyze the underlying mechanisms at both the cellular and network levels. By employing this computational approach, we can identify the critical features involved and optimize stimulation protocols. Additionally, it allows us to test these protocols in silico, functioning as a digital twin for further exploration and refinement.

## 2 Results

**Figure 1:**
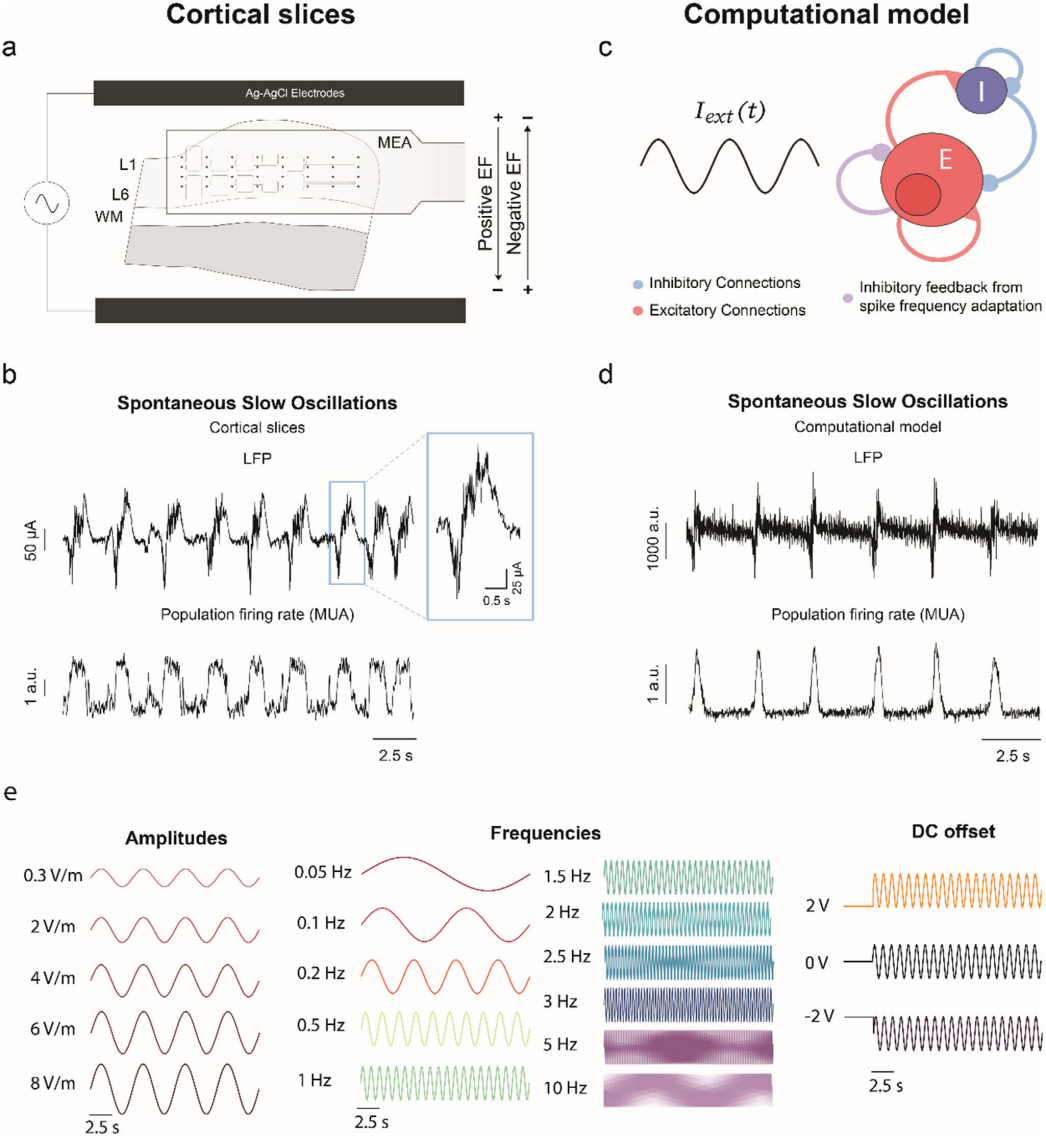
AC stimulation in vitro and in silico. a) AC fields were applied through two Ag‐AgCl electrodes placed parallel to the white matter (WM). Positive fields were defined as those inducing depolarization, oriented from the cortical surface to the WM, whereas negative fields were defined as those inducing hyperpolarization, oriented from the WM toward the cortical surface. Local field potential (LFP) was recorded by resorting to a 32‐channel multielectrode array (MEA) covering multiple cortical columns and layers. b) Slices expressed spontaneous slow oscillations (SO). LFP trace exemplifying the SO activity recorded, with one Up state zoomed in. The LFP was high‐pass filtered at 200 Hz to remove stimulation artefacts. The multi‐unit activity (MUA), which represents an estimation of the population firing rate, was calculated from the filtered LFP. Analysis was performed using the MUA. c) Diagram of the computational model. External stimulation is fed to all populations in a homogeneous way. d) SO activity in the computational model. e) AC fields were applied with amplitudes ranging from 0.3  to 8 Vm^−1^ and frequencies from 0.05  to 10 Hz (not all represented here). Besides AC fields alone (DC offset = 0 Vm^−1^), AC fields were also superimposed over a positive (DC offset = 2 Vm^−1^) or a negative (DC offset = −2 Vm^−1^).

In this study, we recorded from a total of 37 cortical slices expressing spontaneous SO, a rhythm that emerges from the cortical network (**Figure** **1**b). This rhythm consists of Up states, or periods of activity, and Down states, or periods of silence,^[^ ^45^ ^]^ a network activity pattern that we measure as local field potential (LFP) and multiunit activity (MUA), which is an estimation of the population firing rate (see Experimental Section). We investigated the modulation exerted by AC electric fields, with the objective of understanding in detail the interaction between exogenously imposed electric fields and the internal network dynamics. To this end, we used sinusoidal AC fields across a wide range of amplitudes (A; 0.3–8 Vm^−1^) and frequencies ($\tilde{\nu };$ 0.05 –10 Hz) (Figure 1e) with a complete protocol in 16 slices, and in combination with DC in 6 slices. We observed a variety of emergent activity patterns in response to changes in amplitude (A) and relative frequency of stimulation ($\tilde{\nu }$), which can be grouped by regions of the ($\tilde{\nu }$, A) parameter plane displaying the same kind of behavior. Additionally, we investigated how a positive or a negative DC offset affected the response of the network to the AC stimulation. The results were quantitatively reproduced in a computational model, which allowed us to investigate the mechanisms underlying these responses. The network model consists of spiking neurons (Figure 1c) at the level of both spiking activity (i.e., the MUA in Figure 1d‐bottom) and LFPs (Figure 1d‐top), modelled as in Camassa et al.^[^ ^46^ ^]^ Adapted from Torao‐Angosto et al.,^[^ ^47^ ^]^ the model is composed of 9460 leaky integrate‐and‐fire (LIF) neurons (73% excitatory and 27% inhibitory). Synaptic connectivity was tuned to have firing rates in the range of those observed both in vivo and in vitro experiments when cortical neurons display SO (see Experimental Section). Excitatory neurons receive an additional activity‐dependent inhibitory ionotropic current modelling spike‐frequency adaptation, needed to observe relaxation oscillations matching quantitatively SO in cortical networks.^[^ ^48^ ^]^ Stimulations were implemented by injecting into the excitatory neurons an additional external current proportional to the electric field intensity.^[^ ^49^ ^]^ The computational model allowed us to formulate our hypothesis and gain insights into the mechanisms underlying the network response to DC and AC, to eventually test in our experimental preparation.

### 2.1 AC Modulation of Cortical Dynamics is Frequency‐ and Amplitude‐Dependent

The modulation of cortical activity by AC fields depends on the properties of the network and the intrinsic frequency of oscillation ($\omega_{ud}^{0}$) or basal excitability. In our sample, the spontaneous SO frequency average was 0.50 ± 0.06 Hz (mean ± standard error of the mean, n = 16), with values ranging from 0.22  to 1.06 Hz (Figure 1b). However, the effect of AC stimulation on the spontaneous SO also relies on the properties of the stimulation input, such as the frequency (ν) and amplitude (A) of stimulation. Therefore, we will refer hereafter to the relative frequency of stimulation with $\tilde{\nu }=\nu /\omega_{ud}^{0}$. The interaction between the spontaneous SO activity and the AC field was quantified by resorting to the amplitude and phase locking value (APLV, see Methods). High APLV indicates robust synchronization between the perturbed activity and the stimulation signal, while a low APLV indicates a lack of coupling between the detected‐Up states and the stimulation input.

**Figure 2:**
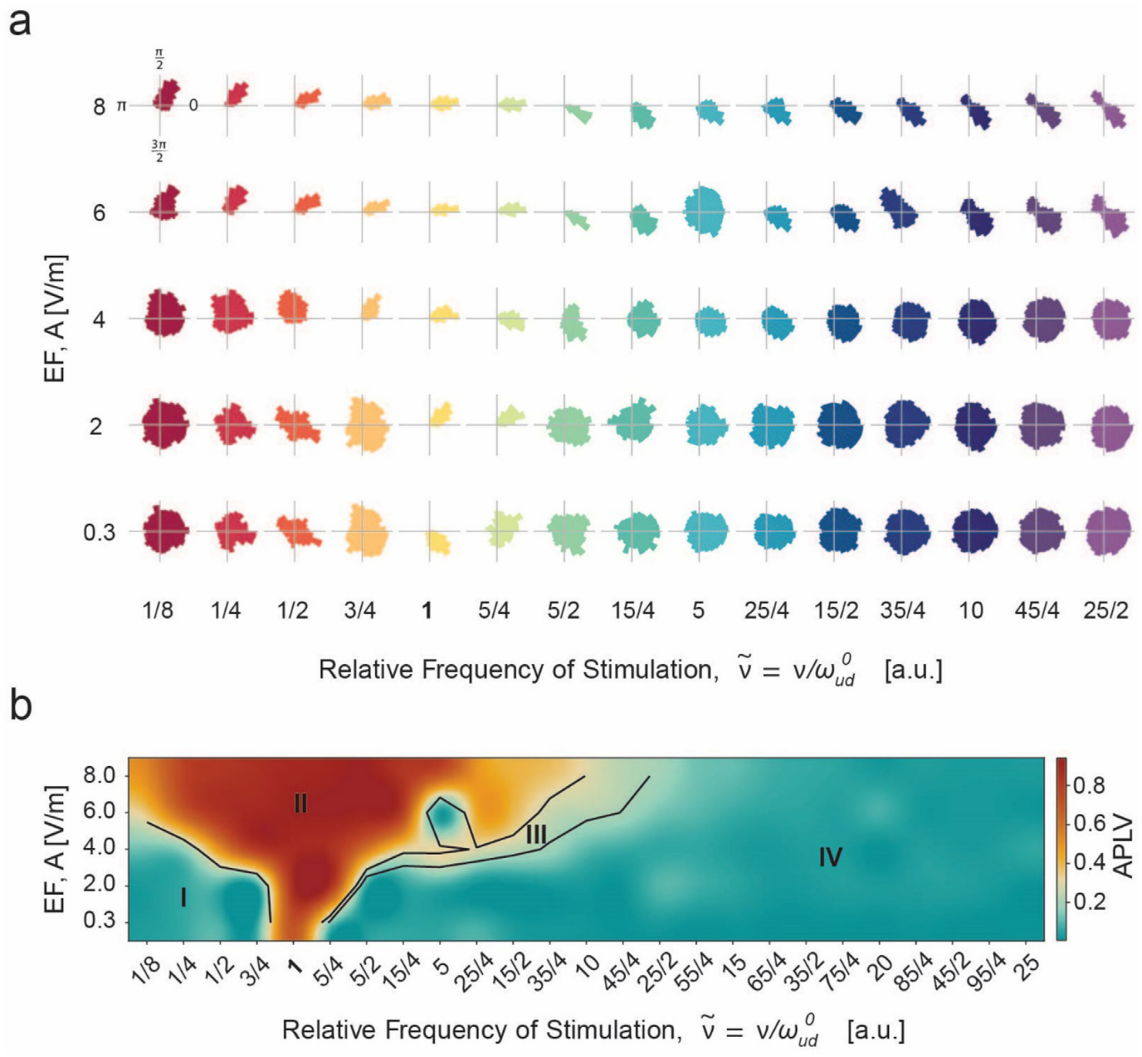
AC modulation of cortical dynamics is frequency‐ and amplitude‐dependent. a) Phase histograms in polar coordinates for different pairs of amplitude and frequency of stimulation. Phase preference is clear at frequencies of stimulation similar to the intrinsic frequency of oscillation ($\omega_{\mathrm{ud}}^{0}\approx 0.4$, $\tilde{\nu }\approx 1$). Phase‐locked region widens with the increase in amplitude. Other regimes can be observed between the phase‐locking and the no phase preference cases. In polar plots, phase differences are expressed in polar coordinates, with angles given in radians: 0, π/2, π, and 3π/2 corresponding to the standard directions on the unit circle. b) Amplitude and phase locking values (APLV) for the different amplitudes and frequencies of stimulation for one representative example. Frequency is normalized to the frequency of the slow wave activity during control (relative frequency of stimulation). An Arnold tongue emerges, centered at the frequency of stimulation closer to the intrinsic frequency of oscillation (relative frequency of stimulation $\tilde{\nu }\approx 1$). Entrainment occurs in the region inside the Arnold tongue. With the increase of amplitude, a wider range of stimulation frequencies can lead to entrainment. A variety of cortical dynamics are observed in the limits of the Arnold tongue and in regions further away from the Arnold tongue.

As depicted in the example in **Figure** **2**, the neuronal response to the different ($\tilde{\nu }$, A) pairs tested gave rise to an Arnold tongue scenario. Mainly, we observed the emergence of a 1:1 Arnold tongue, that is, centered at $\tilde{\nu }=1$. Figure 2a represents the polar plots of the APLV for the different amplitudes and frequencies. The polar plots illustrate how, at 0.3 Vm^−1^ the only phase‐locked response is the one at the intrinsic frequency, expanding with the amplitude of the field up to 8 Vm^−1^, where the responses to all explored frequencies are phase‐locked. The theory suggests that stimulation frequencies near the intrinsic frequency of the network ($\tilde{\nu }\approx 1$) can induce significant locking between the emergent activity and the applied sine wave, even at low amplitudes of stimulation. With a progressive increase in amplitude, the range of stimulation frequencies inducing phase‐locking dynamics and entrainment also expands, forming an Arnold tongue.^[^ ^29^, ^32^ ^]^ As such, the region delimited by the Arnold tongue corresponds to the ($\tilde{\nu }$, A) pairs that lead to the highest APLV.

**Figure 3:**
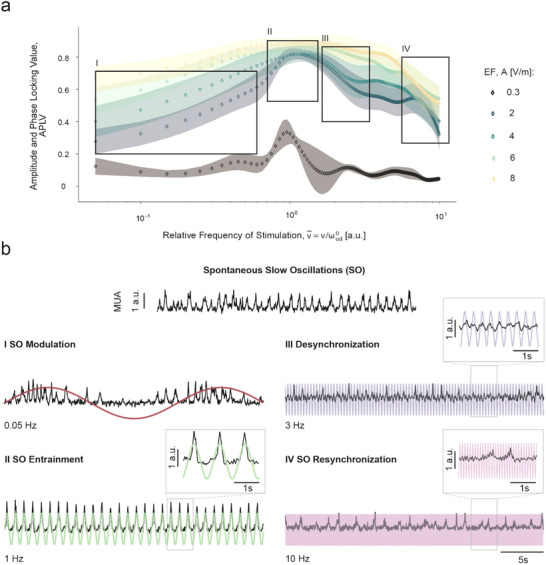
Cerebral cortex dynamics emerging from AC stimulation at different frequencies and amplitudes. a). Average amplitude and phase locking values (APLV) for each amplitude of stimulation (n = 6 for 0,3 Vm^−1^, n = 11 for 2 Vm^−1^, n = 9 for 4 Vm^−1^, n = 12 for 6 Vm^−1^, n = 9 for 8 Vm^−1^) in function of the relative frequency of stimulation. The shaded areas around each curve represent the standard error of the mean (SEM) for each amplitude. At higher amplitudes of stimulation, the APLV values remain high throughout a wider range of stimulation frequencies. At 2  and 4 Vm^−1^, the peak in the APLV values related to entrainment is followed by a steep decrease that later stabilizes. The different regions (I – SO Modulation, II – SO Entrainment, III – Desynchronization, IV – SO Resynchronization) delimitated in the graph relate to different cortical dynamics generated by the interaction of the emergent slow wave activity with the AC stimulation. b). The multi‐unit activity (MUA) traces exemplify some of the dynamics observed in these regions by different frequencies at a fixed amplitude of 2 Vm^−1^, as well as the control activity (intrinsic frequency of 1.06 Hz). SO Modulation (I) occurs at frequencies lower than the intrinsic frequency, with Up states preferentially appearing during the positive part of the sine wave. In this case, the oscillatory frequency does not match the frequency of stimulation. At frequencies close to the intrinsic frequency, entrainment is clear (II), with the frequency of oscillation shifting to match precisely the frequency of stimulation. At the frontier of the Arnold tongue, entrainment disappears and gives place to a decrease in the APLV value (III). In this region, the Up/Down dynamics are lost and there is a high variability in the amplitude of the signal. At frequencies further away from the intrinsic frequency (IV), activity restores characteristics from the control activity.

However, what is the response of the cortical network to ($\tilde{\nu }$, A) pairs outside the Arnold tongue region? Here, we describe for the first time experimentally a range of complex cortical dynamics that result from the interaction between the spontaneous SO activity of the local cortical network and exogenous AC fields at different points in the APLV ($\tilde{\nu }$, A) plane (Figure 2b). Based on the emerging Arnold tongue, we were able to define four different regions in which the resulting neuronal response exhibited similar behavior: I – SO modulation; II – SO Entrainment; III – Desynchronization; and IV – Resynchronization. These regions can be seen in Figure 2b for one case example. However, the Arnold tongue scenario was present in all slices studied, as depicted in **Figure** **3**, where the average APLV for all cases is shown.

### 2.2 Network Desynchronization with Sine Waves: from Synchrony to Asynchrony by Periodic Stimulation

The different AC stimulation parameters applied revealed a range of dynamic responses, which are translated by changes in the APLV. The average APLV for each amplitude of stimulation (*n* = 6 for 0.3 Vm^−1^, *n* = 11 for 2 Vm^−1^, *n* = 9 for 4 Vm^−1^, *n* = 12 for 6 Vm^−1^, *n* = 9 for 8 Vm^−1^) in a function of the relative frequency of stimulation is depicted in Figure 3a. At the lowest amplitude tested (0.3 Vm^−1^), an increase in APLV only occurred at frequencies of stimulation close to $\omega_{ud}^{0}$ ($\tilde{\nu }$ ≈ 1) and this peak was much lower when compared with other amplitudes, which indicates a weaker coupling between the stimulation signal and the cortical activity. At higher stimulation amplitudes, the APLV values remained high throughout a wider range of stimulation frequencies. At 2  and 4 Vm^−1^, the peak in the APLV values was followed by a steep decrease that later stabilized. For 6  and 8 Vm^−1^, the system seamlessly locked in with the driving force until $\tilde{\nu }$ ≫ $\omega_{ud}^{0}$. More specifically, we can describe each experimentally obtained collective behavior using its frequency and phase properties:

#### 2.2.1 SO Modulation

In this region, located to the left of the Arnold tongue in the parameter plane ($\tilde{\nu }$, A) Up states preferentially appeared during the depolarizing portion of the sinusoidal wave, but the intrinsic frequency of the network did not match the frequency of stimulation (0.05 Hz example in Figure 3b).

#### 2.2.2 SO Entrainment

**Figure 4:**
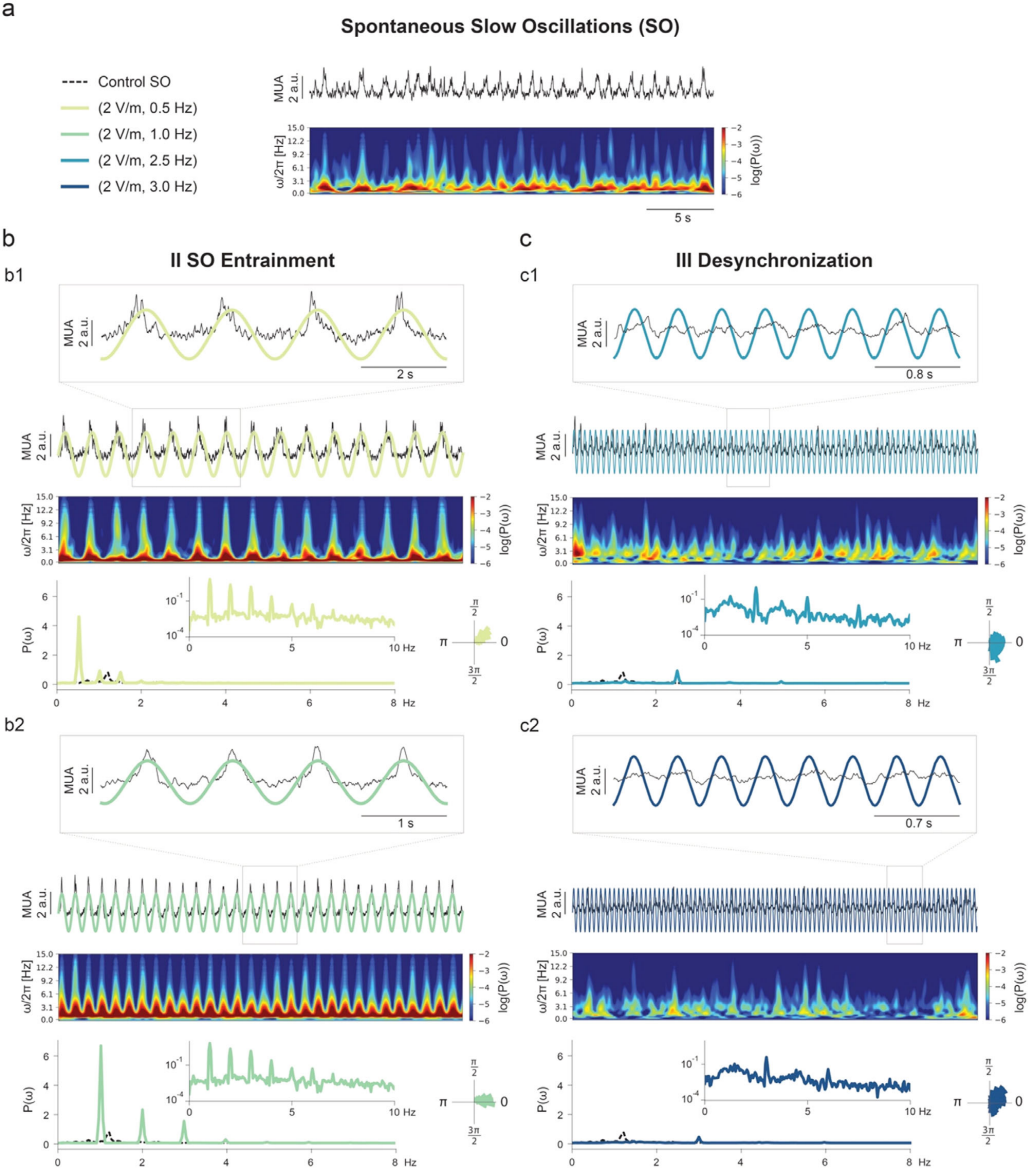
Periodic stimulation can enhance (entrain) or quench (desynchronize) oscillations, depending on the amplitude and frequency of stimulation. a) Control activity with the respective spectrogram. The spectrogram depicts the logarithm of the power of each frequency (ω) at a specific time point. The control power spectrum density (PSD, P(ω)) trace (dotted black line), showing a peak at the intrinsic frequency, is also included in the remaining PSD plots (b. and c.) for comparison purposes. All examples of response to stimulation (b1., b2., c1. and c2.) display their respective multi‐unit activity (MUA) traces, spectrogram, PSD, and phase histogram (polar plot). b) Entrainment occurs at frequencies of stimulation close to the intrinsic frequency. Example responses for a stimulation frequency of 0.5  and 1 Hz can be seen in b1. and b2., respectively. In these cases, the PSD displays clear peaks at the frequency of stimulation and its harmonics. c) In region III, where desynchronization occurs, there is a flattening of the PSD, with high variability in the amplitude and phase of the signal. During stimulation, cortical activity shifts from the slow wave state toward awake‐like activity. Example responses for a stimulation frequency of 2.5  and 3 Hz can be seen in c1. and c2., respectively.

Entrainment, observed within the Arnold tongue region, is characterized by the highest APLV. When entrainment occurred, the network's oscillations were synchronized with the stimulation signal, with the frequency of oscillation shifting to match the frequency of stimulation (1 Hz example in Figure 3b, 0.5  and 1 Hz examples in **Figure** **4**b). Power spectra in this region revealed a dominant peak at the stimulation frequency, accompanied by periodic peaks at its harmonics (Figure 4b). This synchronization is also marked by a constant phase difference over time, indicative of coherent neural activity entrained to the external rhythm.

#### 2.2.3 Desynchronization

As stimulation parameters approached the right boundary of the Arnold tongue, entrainment dissipated, leading to more complex dynamics and eventual desynchronization (3 Hz example in Figure 3b, 2.5  and 3 Hz examples in Figure 4c). In this region, both the MUA amplitude and the phase preference displayed higher variability, which resulted in a decrease in the APLV average value (Figure 3a), with a steeper decay for the amplitudes of 2  and 6 Vm^−1^. This region is characterized by the transition to a desynchronized state, with suppression of the SO. The resulting oscillations showed lower amplitude and higher frequencies, similar to those observed during wakefulness (Figure 4c).

Within this region, we detected different stages toward desynchronization. First, we identified the emergence of quasi‐periodic states, characterized by: 1) an amplitude variation in the response, 2) the presence of incommensurate frequency in the power spectrum (i.e., not harmonically related) and 3) the absence of a consistent preferred phase difference (1 Hz, AC 2 Vm^−1^ example in Figure 6c). By further increasing $\tilde{\nu }$, the system transitioned into the desynchronized state, where neither phase nor amplitude was kept constant (3 Hz example in Figure 3b, 2.5 and 3 Hz examples in Figure 4c). In this state, there was a significant reduction in the power of the spectral density when compared with entrainment (by approximately one order of magnitude), leading to a nearly flat spectrum, indicative of desynchronized activity (Figure 4c). Accordingly, we observed a suppression of the SO, with a loss of Up/Down dynamics, and the emerging activity was no longer synchronized with the stimulation signal.

#### 2.2.4 SO Resynchronization

**Figure 5:**
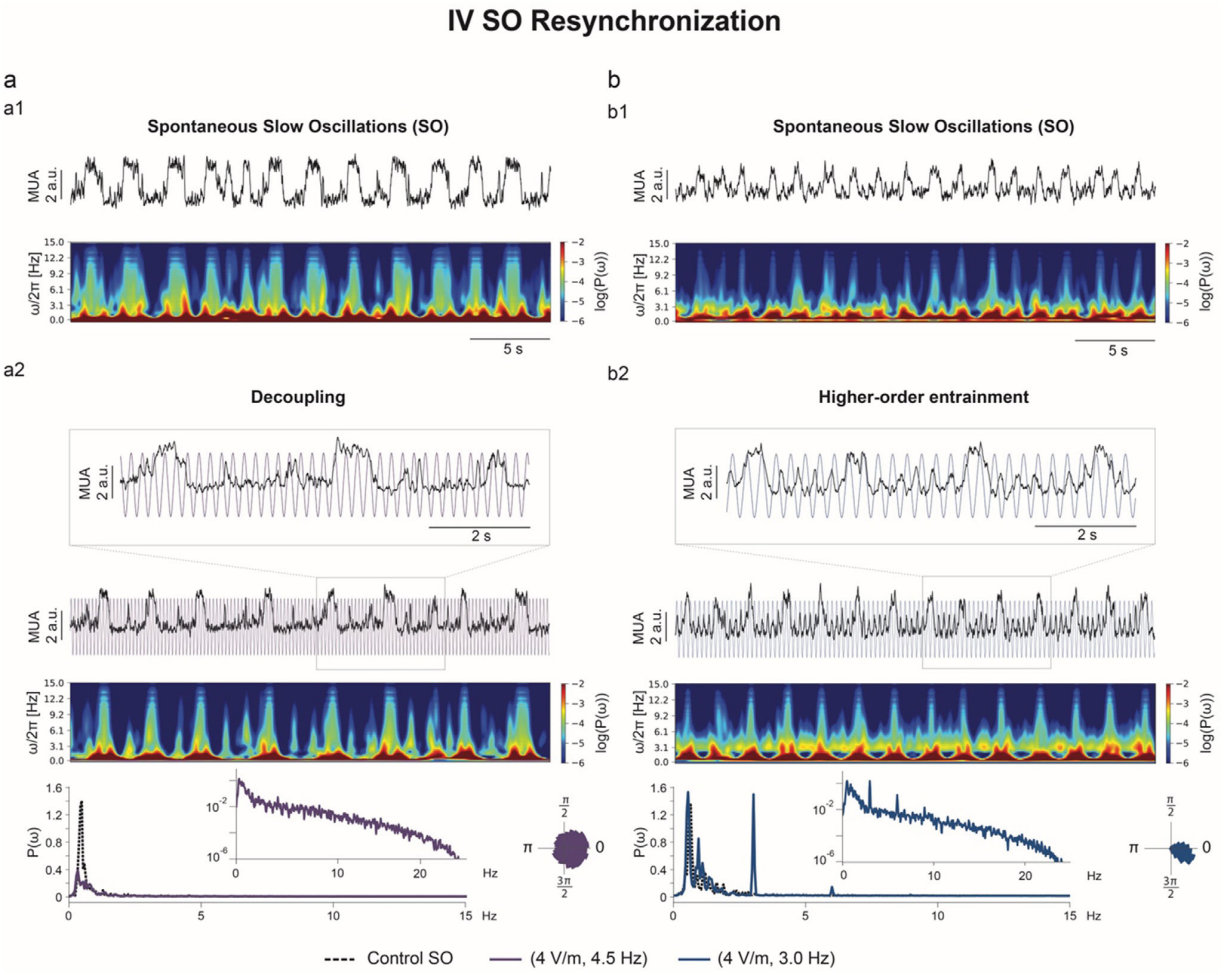
Synchronicity resumes at frequencies of stimulation further away from the intrinsic frequency. Within region IV – SO Resynchronization, SO activity is resumed, either decoupled from the stimulation input (a.) or following a higher‐order entrainment regime (b). For both cases, the multi‐unit activity (MUA) traces, spectrogram, and power spectrum density (PSD, P(ω)) are depicted for the respective control activity (a1. and b1.) and for the stimulation period (a2. and b2.). In the case of decoupling (a), the PSD from the stimulation period follows the PSD from control, but with lower amplitude, and the phase histogram shows no phase preference. During higher‐order entrainment (b), besides following the PSD from control, an almost equal peak is also seen at the stimulation frequency in the stimulation PSD, followed by a smaller peak at its harmonic. The phase histogram reveals a phase preference.

As stimulation parameters ($\tilde{\nu }$, A) moved further away from the ones included in the Arnold tongue ($\tilde{\nu }$ ≫ 1), synchronized activity resumed. Depending on the specific ($\tilde{\nu }$, A), a range of dynamics within the SO regime were observed, including decoupling (4.5 Hz example in **Figure** **5**a) and higher‐order entrainment (3.0 Hz example in Figure 5b). Decoupling occurred preferentially at lower amplitudes of stimulation and emerged when the local network was not driven by the stimulation signal. This state is characterized by a power spectrum that resembles the one during baseline and by no relative phase preference. When decoupling was observed, the system remained in this steady state regardless of further increases in the frequency of stimulation. In some cases, the frequency of oscillation of the emerging activity during stimulation matched the baseline frequency ($\omega_{ud}=\omega_{ud}^{0}\;$). In other cases, stimulation resulted in the appearance of a synchronized activity with Up/Down state dynamics, which displayed a frequency of oscillation different from the intrinsic frequency of the system ($\omega_{ud}\neq \omega_{ud}^{0}$). Conversely, higher‐order entrainment, observed at higher amplitudes of stimulation, manifested as two co‐existing frequencies in the power spectrum: one corresponding to the intrinsic network frequency and the other denoting resonance with the stimulation frequency. Besides, the instantaneous phase difference remained constant (Figure 5b). The region of higher‐order entrainment is represented in the APLV parameter plane in Figure 3a by a second peak at higher frequencies that has a lower amplitude than the entrainment peak.

The question now arises: How can we precisely attain each of these dynamics? What is the correct combination of parameters ($\tilde{\nu }$, A)? In this section, we have qualitatively demonstrated the different dynamics that can emerge with this type of stimulation; however, the suppression of the SO was not always consistently achieved. We considered that designing a more robust protocol that guaranteed not only the entrainment but also the desynchronization was needed.

### 2.3 Designing a Robust Protocol: Integrating AC and DC to Effectively Induce Entrainment and Desynchronization

**Figure 6:**
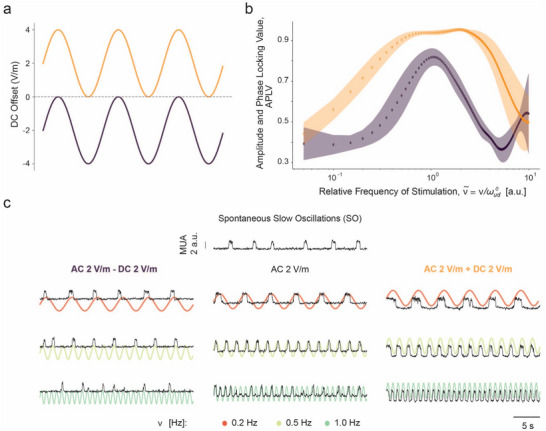
Effect of introducing a DC offset in the emerging cortical dynamics. a) Scheme of the applied AC fields: an AC field of 2 Vm^−1^ was superimposed over either a positive DC field of 2 Vm^−1^ (yellow, *n* = 6) or a negative DC field of 2 Vm^−1^ (purple, *n* = 6). b) Amplitude and phase locking values (APLV) as a function of the relative frequency of stimulation for the two DC offsets used. The shaded areas around each curve represent the standard error of the mean (SEM) for each amplitude. While a positive offset increases the range of frequencies able to result in a high APLV value (entrainment region), the negative DC offset widens the range of stimulation frequencies, leading to a decrease in synchronization (toward desynchronization region). c) Example MUA traces for the control (top) and the resulting dynamics when the same three differences were applied (0.2 , 0.5 , and 1 Hz) at three different DC offset levels: AC field of 2 Vm^−1^ with a negative DC offset of 2 Vm^−1^, AC field of 2 Vm^−1^ without a DC offset and AC field of 2 Vm^−1^ with a positive DC offset of 2 Vm^−1^. The same frequency can result in different cortical dynamics depending on the DC offset used. For example, while 1 Hz stimulation leads to activity with low synchronization with the negative DC offset, no offset results in a quasiperiodic state, and in the positive offset, entrainment is maintained.

We observed that, by applying DC fields, we could more easily modulate the emerging dynamics and achieve more robust results. We applied the same sinusoidal stimulation protocol (A = 2 Vm^−1^, ν  = 0.05–10 Hz) with a DC offset of −2  or 2 Vm^−1^ (*n* = 6) to explore how the change in excitability created by the DC field would affect the different dynamics enabled by the AC fields. The negative DC offset led to a shortening of the range of stimulation parameters, leading to entrainment, with more ($\tilde{\nu }$, A) pairs leading to desynchronization. Contrarily, a positive DC offset vastly extended the entrainment range beyond $\tilde{\nu }$ = 1, avoiding desynchronization. As can be seen in **Figure** **6**, this effect of the DC offset was translated by a narrow APLV peak for the negative DC offset, followed by a steep decrease that extended for a wide range of frequencies. In the case of the positive DC offset, the average APLV was maintained at its maximum for most of the frequencies tested, forming a plateau. The value of average APLV during this plateau (Figure 6b) was even larger than the value of APLV reached even for the largest fields (8 Vm^−1^) without DC (Figure 3a). However, for the negative DC offset (Figure 6b), the peak remained at similar amplitudes to those without DC (Figure 3a). This suggests that within the ($\tilde{\nu }$, A) parameters tested, maximum amplitude and phase locking are achieved when a positive DC offset is applied, even at a lower A. As such, when keeping a constant A = 2 Vm^−1^, the same $\tilde{\nu }$ can lead to the emergence of distinct cortical dynamics, depending on the DC offset being applied. In the example in Figure 6c, while at ν = 0.2 Hz, all DC offsets led to entrainment, at ν = 0.5 Hz entrainment was no longer achieved with the negative DC offset, and at ν = 1.0 Hz entrainment only occurred with the positive DC offset. Contrarily, desynchronization started earlier when the negative DC offset was applied (half‐harmonics at ν = 0.2 Hz and desynchronization at ν = 1.0 Hz) when compared with when no DC offset was applied (quasi‐periodicity at ν = 1.0 Hz). Therefore, the superimposition of a negative and positive DC offset over the AC field enabled more robust and extended regions of desynchronization and entrainment, respectively.

### 2.4 A Cortical Network Model for the Dynamical Investigation of Sinusoidal Modulation

Our experimental observations shed light on the emergence of diverse qualitative collective behavior during AC stimulation, including phase and frequency locking, quasi‐periodicity, and desynchronization. However, for a detailed mechanistic understanding of cellular and network AC‐intrinsic rhythm interaction, we resorted to a model of the cortical network composed of excitatory and inhibitory spiking neurons capable of reproducing the spontaneous activity of cortical slices (see Experimental Section). Hence, we explored whether time‐varying stimulations modulating the external current could reproduce the collective response observed in experiments.

**Figure 7:**
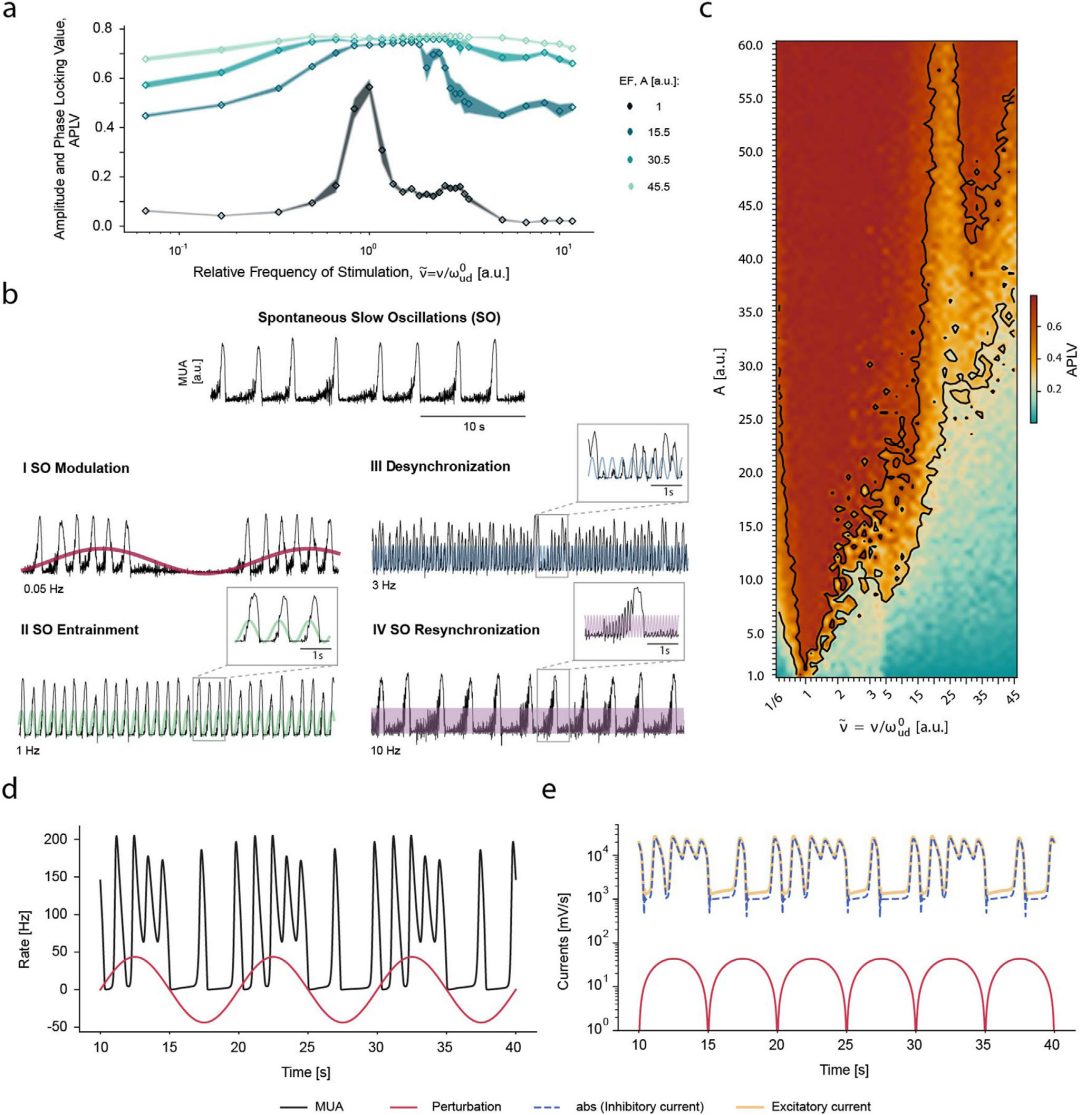
Cortical dynamics emerging from AC stimulation are reproduced in a computational model. a) APLV value at different amplitudes of stimulation for a range of different relative frequencies of stimulation. The shaded areas around each curve represent the standard error of the mean (SEM) for each amplitude. b) MUA traces exemplifying the spontaneous slow oscillations (SO) generated by the model and the model's equivalents for the four dynamical response regions detected in the experimental data: I – SO Modulation, II – SO Entrainment, III – Desynchronization, IV – SO Resynchronization. c) The model predicts the emergence of several different Arnold tongues at harmonics of the intrinsic frequency, which represent regions of higher‐order entrainment. Rate (d.) and currents (e.) from the neuronal activity in comparison to the perturbation. The inhibitory and excitatory currents generated by the neurons are much higher when compared with the currents generated by the perturbation.

As can be seen in **Figure** **7**a,b, the model was able to reproduce the same regions detected experimentally. Moreover, by being able to explore a wider range of stimulation parameters, it was possible to observe in the model the emergence of several Arnold tongues of a different order; that is, at stimulation frequencies that are multiples of the spontaneous rhythm of the network (Figure 7c), giving rise to the higher‐order entrainment also observed in cortical slices. Intriguingly, we found that even relatively small changes at the microscopic level in neuronal excitability can be amplified by the nonlinear and collective dynamics of cortical networks, giving rise to a rich repertoire of cortical dynamics (Figure 7d,e).

### 2.5 Properties of the Desynchronized Cortical Network: an In Vitro and In Silico Mechanistic Analysis

**Figure 8:**
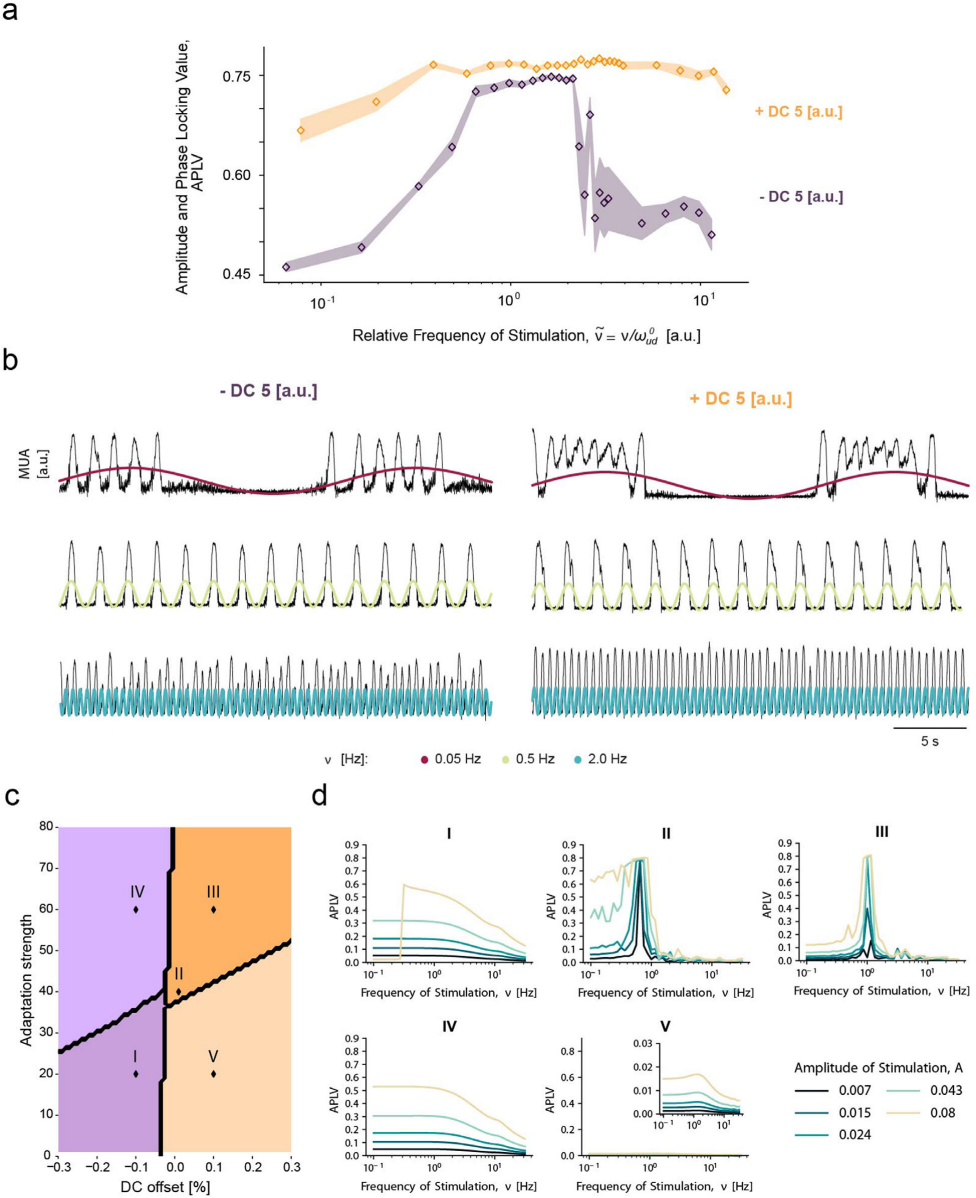
A computational model of nonlinear oscillators can reproduce the effect of a DC offset on the range of frequencies leading to entrainment (positive DC offset) or desynchronization (negative DC offset). a) Amplitude and phase locking values (APLV) for a range of relative frequencies of stimulation for a positive (yellow) and a negative (purple) offset. The shaded areas around each curve represent the standard error of the mean (SEM) for each amplitude. b) MUA traces exemplify the emergence of modulation at 0.05 Hz and entrainment at 0.5 Hz for both offsets. However, at 2.0 Hz, the negative offset leads to a decrease in the synchronization of the network, and with a positive offset, entrainment is still clear. c) Adaptation strength versus external DC current bias bifurcation diagram. (*I*–*V*) represent different points in the plane, which are associated with different dynamical regimes. d) These regimes are translated by the APLV profiles calculated for a range of frequencies and amplitudes of stimulation.

The effect of adding a DC offset to the AC stimulation in the emerging cortical dynamics could also be reproduced in the computational model (**Figure** **8**a,b). To better understand the response of the network, it is instructive to look at the (Excitation, Adaptation) bifurcation diagram obtained by varying the adaptation strength and the external DC current bias (excitation) (Figure 8c). Four different dynamical regimes were found according to mean‐field theory:^[^ ^48^, ^50^, ^51^ ^]^ a high‐firing asynchronous state (phase V); a low‐firing asynchronous state (phase IV); a bistable regime where both are present (phase I) and finally a regime of SO (phase including case II and III) where the activity of the network is synchronized with a frequency that depends mostly on the (Excitation, Adaptation) parameters. When an AC stimulus is applied, the system moves in time in the bifurcation plane, and consequently, it can explore different dynamical regimes during different phases of the stimulation. The rich repertoire of response patterns we have observed looking at the APLV at different stimulation amplitudes and frequencies, both in the model and in the slices, is a consequence of this state‐dependent modulation of the intrinsic properties of the systems (Figure 8d). Cortical slices are, in fact, nonlinear (relaxation) oscillators^[^ ^48^, ^52^ ^]^ with a proper frequency and activity amplitude. While this complicates the analysis with respect to a linear oscillator, this kind of nonlinear system, when periodically forced, can give rise to nontrivial entrainment phenomena such as parametric resonance and Arnold tongues^[^ ^53^ ^]^ that we observed in both the model and experiments. One could argue that these peculiar response patterns might arise only in cases where the strength of the stimulation is so high that it is essentially dominant with respect to the rest of the synaptic current. However, our mean‐field model demonstrates that this is not the case. In fact, this peculiar response pattern can be achieved with an external current that is just 10% of the total synaptic current (Figure 7e). This phenomenon can be explained by the nonlinear amplification of the recurrent excitation competing with the inhibitory feedback due to adaptation. The nonlinear amplification gives rise to a nonlinear relaxation oscillator, capable of resonating with weak external perturbations.

## 3 Discussion

Electrical stimulation targeting brain oscillations not only provides useful insights into fundamental physiological processes but also facilitates advances in neurostimulation clinical applications.^[^ ^24^ ^]^ Here, our aim was to explore the interaction between spontaneous cortical oscillations and an exogenous sinusoidal perturbation. To accomplish this, we applied AC fields with a wide range of amplitudes (0.3 to 8 Vm^−1^) and frequencies (0.05  to 10 Hz) to cortical slices in vitro expressing spontaneous SO. Additionally, we explored the influence of a DC offset on the emerging dynamical responses. In addition to the well‐described entrainment at stimulating frequencies close to the endogenous network frequency,^[^ ^28^, ^33^, ^54^, ^55^, ^56^ ^]^ a rich repertoire of responses could be described at different combinations of amplitude, frequency, and DC offset, including desynchronization, high‐order entrainment, and decoupling. Lastly, we explored the mechanisms behind the dynamical cortical response to a sinusoidal perturbation, resorting to a computational model. Our findings significantly improve the understanding of how external electric fields influence cortical network dynamics, with implications for both therapeutic and experimental applications in neuroscience.

To gain a comprehensive understanding of the system's dynamics and achieve precise control over the stimulation parameter plane, we created a detailed map of possible emerging dynamics based on the system's intrinsic properties and external driving parameters. We accomplished this by measuring the APLV, which revealed an Arnold tongue scenario that helped us identify the different possible collective behaviors that could emerge within the driving parameter plane. Specifically, we observed an Arnold tongue centered at the endogenous frequency, consistent with previous studies suggesting that tACS can effectively entrain network oscillations when applied at frequencies close to the endogenous oscillation frequency.^[^ ^27^, ^28^, ^29^, ^31^, ^32^ ^]^ However, besides the well‐described entrainment region,^[^ ^28^, ^33^, ^54^, ^55^, ^56^ ^]^ other more complex, nonlinear cortical responses emerged outside the boundaries of the Arnold tongue.

We have described several regions with respect to the network's response to AC stimulation (see Results). In both region I (SO Modulation) and region II (SO Entrainment), the network exhibited a stable periodic behavior, where each cycle was approximately a reproduction of the previous cycle. The same does not apply to the desynchronized state (region III), occurring at the right boundary of the Arnold tongue, in which there was a large variability in response to the sinusoidal input, resulting in a loss of periodicity. While the activity within the desynchronization region did not necessarily match the asynchronous state characteristic of wakefulness, some features resembled awake‐like activity. This regime was characterized by activity with low amplitude, very high variability, no phase locking, and a flattening of the power spectrum. The transition from synchronized to desynchronized states under sinusoidal perturbations emphasizes the nonlinear nature of cortical network responses to external stimulation. This nonlinearity likely arises from the intrinsic properties of the network, such as the balance between excitatory and inhibitory synaptic reverberation, which can amplify or dampen the effects of external stimuli. Understanding these nonlinear regimes of the dynamics is essential for developing more precise and effective neuromodulation strategies.

To our knowledge, this is the first time that desynchronization induced by AC stimulation has been reported experimentally. A recent experimental study reported that AC stimulation does not always lead to an increase in the entrainment of the network.^[^ ^57^ ^]^ Single‐neuron recordings in the nonhuman primate brain, aiming to characterize the interaction between ongoing oscillations and tACS, revealed that entrainment only occurred when neural firing was independent of ongoing oscillations. Contrarily, when neural activity was strongly driven by endogenous brain oscillations, tACS resulted in a decrease in entrainment. This reduction could only be overturned with higher stimulation amplitudes.

Our observations align with predictions from computational models, which proposed that, when stimulated with AC fields, nonlinear dynamics could emerge outside the Arnold tongue.^[^ ^29^, ^58^, ^59^, ^60^ ^]^ Based on models, Ali et al.^[^ ^29^ ^]^ suggested that, for stimulation parameters outside the Arnold tongue, epochs of stable entrainment alternate with periods of desynchronized activity. According to this model, the relative time interval spent on entrained versus non‐entrained activity would then depend on the amplitude of stimulation and on the distance between the intrinsic frequency and the frequency of stimulation. Strong suppression of cortical oscillations occurred for frequencies of stimulation between the dominant endogenous frequency and its first harmonic frequency. In our experimental results, we observed that the desynchronized region occurred at stimulation frequencies higher than those leading to entrainment. The effect of stimulating at the harmonic frequencies depended on the amplitude of stimulation, with harmonic frequencies leading to higher‐order entrainment at higher amplitudes of stimulation.

Our results highlight that both the amplitude and frequency of external stimulation are critical in shaping the network's response. This aligns with previous studies emphasizing the frequency‐dependent effects of tACS on neural synchronization.^[^ ^27^, ^28^, ^29^, ^54^, ^61^, ^62^ ^]^ However, our work extends these insights by demonstrating that amplitude variations also play a crucial role in determining the network's collective behavior. We observed that higher amplitudes can induce higher‐order entrainment ($\tilde{\nu }\gg 1$, Region IV – SO Resynchronization), where more complex synchronization patterns emerge, such as phase‐locking, where the forcing frequency aligns with rational multiples of the network's natural frequency. The occurrence of entrainment at the harmonics of the intrinsic frequency has also been previously suggested by computational models.^[^ ^28^, ^29^ ^]^

Here, we have described a nonlinear interaction between the local cortical network and weak AC fields. As such, the same network can exhibit distinct dynamical responses when stimulated with AC fields at different pairs of amplitude and frequency. Consequently, it highlights the importance of utilizing the appropriate stimulation parameters to obtain a specific response. Considering that the fields applied in the clinic are usually ≤  1 Vm^−1^, it is especially valuable to stimulate with frequencies very close to the target intrinsic oscillation to obtain entrainment, as we have observed that, for the lowest amplitudes, entrainment is only achieved for a narrow range of frequencies around the intrinsic one. This could also explain why there are so many contradictory reports concerning the efficacy of tACS stimulation in the modulation of cortical dynamics, as often the frequency of stimulation is chosen as an estimation of the peak of the desired frequency band. Since the dominant frequency within a target frequency band can vary across individuals^[^ ^63^, ^64^ ^]^ or even within the same individual at different time points,^[^ ^65^, ^66^, ^67^ ^]^ stimulating with a fixed frequency can lead to contrasting results, leading to either an enhancement or a reduction of the target pattern. Our results indicate that a frequency of stimulation above the intrinsic one ($\tilde{\nu }>1)$ drives the network to region III of the ($\tilde{\nu }$, A) plane, leading to suppression instead of enhancement of a target oscillation. Besides, desynchronization in our experimental setup was not always achieved since it required rather specific stimulation parameters. Consequently, targeting the entrainment or desynchronization of precise brain oscillations non‐invasively is non‐trivial, which might help explain the high variability reported in human tACS studies. As such, there is a need for developing tACS protocols that are more robust in achieving the target therapeutic response.

While the majority of the current human tACS studies use open‐loop approaches, applying waveforms of a fixed frequency at arbitrary times,^[^ ^57^ ^]^ some studies resort to a closed‐loop approach to stimulate with the most effective frequency and phase. However, combining electrical stimulation with brain signal readouts such as EEG or MEG/MRI is very hard to achieve due to electrical artefact constraints.^[^ ^68^ ^]^ Here, we propose an open‐loop methodology that can increase the effectiveness of a neuromodulation protocol in a rather simple manner. Our results suggest that applying a positive or negative DC offset to an AC field can widen the range of frequencies leading to entrainment or desynchronization at a given intensity, respectively. AC stimulation with a DC offset has previously been applied in the clinic in a limited number of studies and is known as otDCS.^[^ ^27^, ^69^ ^]^ While AC fields mainly affect the temporal structure of the neural activity, DC stimulation is associated with shifts in the transmembrane voltage, which affect the probability of action potential firing.^[^ ^33^ ^]^ These changes in neuronal polarization are compartment‐specific, and different models have explored the contribution of dendrites, somas, and axons,^[^ ^70^, ^71^, ^72^, ^73^ ^]^ the effects being highly dependent on the neuronal morphology and position in the cortical layers. Consequently, DC stimulation can modulate the excitability of the network in a polarity‐dependent way, with excitability being increased by positive fields and decreased by negative fields.^[^ ^74^, ^75^, ^76^, ^77^, ^78^, ^79^ ^]^ otDCS incorporates elements of both DC and AC, concurrently modulating the potential and oscillation activity of neuronal membranes.^[^ ^27^, ^69^ ^]^ As a result, the action of otDCS will also be polarity‐dependent, in line with our results, with fields oriented inward (positive, from the cortical surface to the white matter) leading to an increase in excitability which facilitates entrainment, and the opposite direction (negative, from the white matter to the cortical surface) enabling desynchronization.

By providing a more robust protocol for the achievement of entrainment or desynchronization, the application of a DC offset can have relevant clinical applications. These offsets can be introduced into tACS protocols—positive offset if the goal is the entrainment of the network to a certain frequency, and negative offset if the goal is to desynchronize a hyper‐synchronized pathological oscillation. By facilitating a wider range of frequencies to achieve the same targeted dynamical response, the DC offset could diminish the high variability previously reported in the clinic for tACS applications, improving the outcomes of treatments employing tACS. For instance, tACS with a negative offset may be particularly relevant for patients with drug‐resistant epilepsy – who represent approximately one‐third of all epilepsy cases^[^ ^79^ ^]^ – since epilepsy is characterized by a combination of hyperexcitability and hyper synchronicity of the network.^[^ ^80^, ^81^ ^]^ Besides epilepsy, other neuropsychiatric disorders associated with an increased neural synchronization, such as Parkinson's disease^[^ ^39^ ^]^ and major depressive disorder,^[^ ^82^ ^]^ could also potentially benefit from this approach. Moreover, tACS with offsets could aid in disorders with altered rhythms or synchronization, like schizophrenia,^[^ ^38^, ^83^ ^]^ hyper‐synchronized perilesional cortical areas after brain injury,^[^ ^43^ ^]^ autism spectrum disorder,^[^ ^39^ ^]^ and Alzheimer's disease,^[^ ^84^ ^]^ by enhancing or dampening specific oscillations.

To get a further insight into the interaction between the local cortical network and the exogenous AC fields, we resorted to a computational model, consisting of a network of excitatory and inhibitory spiking neurons. Despite its simplicity, the model accurately replicated the experimentally observed collective behavior. Indeed, although in our model no spatial structure was incorporated, firing patterns simulated in response to the same stimulations adopted in experiments display a remarkably similar behavior. This is in line with the evidence that spatially organized networks like Landau‐Ginzburg (LG) models, previously introduced in di Santo et al. (2018),^[^ ^85^ ^]^ display a bifurcation diagram almost indistinguishable from the one derived under mean‐field approximation in the network of excitatory and inhibitory integrate‐and‐fire neurons.^[^ ^48^, ^50^ ^]^ It is then not surprising to note that LG models, Arnold tongues, and other nonlinear phenomena^[^ ^86^ ^]^ emerge in a similar way as in our simple excitatory‐inhibitory network. Our modeling approach thus offers a faithful physical representation of the cortical slice, predicting the behavior of a structured cortical network in response to time‐varying perturbations. One advantage of its simplicity is that it enables a more extensive exploration of the ($\tilde{\nu }$, A) parameter plane, thereby encompassing a wider range of interactions. Consequently, the model can predict the network response to ($\tilde{\nu }$, A) configurations that were not tested experimentally. In the presented framework, we proved that this modelling approach allows for the design of an effective digital twin of the probed cortical network. Despite its simplicity at the microscopic level of single cells (including only integrate‐and‐fire neurons), the collective dynamics of our model are an effective description of the macroscopic cortical networks, like those investigated in vitro in our study, according to the adage “more is different” by Philip W. Anderson.^[^ ^87^ ^]^

Additionally, the model provided information about the current that the neurons receive through synaptic input, which we did not measure experimentally. This current was much smaller (≈10%) when compared with the input current generated by the applied exogenous fields. However, despite being a weak perturbation, synaptic inputs could affect the dynamics of the neuronal network. This emergent phenomenon is only possible due to a nonlinear amplification of the weak perturbation by the reverberation of the activity that the recurrent network spontaneously generates. This amplification allows the emergence of a rich repertoire of collective behavior. The fact that the perturbation is relatively small implies that the variability observed in the desynchronized state (region III), in response to the same input, results from the stochastic and nonlinear dynamics of the network activity, rather than the stimulation itself. As such, the network displays a high computational capacity, being able to transform a trivial input, as a sinusoidal field, into a complex cortical dynamic.

Taken together, our results suggest that the interaction between the endogenous activity and the exogenous input is nonlinear. When interacting with the brain by exogenous interventions, it is fundamental to consider the internal dynamics of the system. These results align with the hypothesis of brain state‐dependent effects of brain stimulation.^[^ ^88^ ^]^ Moreover, our results indicate that a simple exogenous intervention, provided that the parameters of stimulation are appropriately tuned, can push the brain network into very different dynamical states. Therefore, it is possible to exploit the brain's complexity without the need to design complex exogenous interventions. Finally, the interaction between our local network and the weak AC field can be extrapolated to other systems with interacting oscillators. For example, this interaction could be a model for the interplay between a specific brain region and an internal input from a different brain region or an external input from the outside world (e.g., visual, audio). Furthermore, these principles may be applicable to other existing oscillators, for instance chemical oscillators, such as those observed in the Belousov‐Zhabotinsky reaction, demonstrating how simple local features and interactions can generate complex rhythmic patterns, closely resembling emergent neural and physiological rhythms.^[^ ^89^ ^]^ Like reaction‐diffusion waves in chemical systems, slow waves propagate across the cortex as traveling wave packets, displaying structured dynamics similar to spiral waves emerging from chemical oscillators.^[^ ^90^ ^]^ These chemical reaction networks are capable of reproducing nonlinear relationships as well as oscillatory, chaotic, and excitable regimes, similar to the ones described here. As such, our results may offer valuable insights for neuromorphic engineering, particularly in the context of wetware computing, by providing guiding principles for the development of adaptive, self‐organizing computational platforms at the interface of biology and technology.^[^ ^91^ ^]^

The translation of these findings to clinical applications is not straightforward. First, the current intensity used in clinical studies is limited to ensure the safety of the procedure (≈2 mA), which results in fields with amplitudes much lower (<1 Vm^−1^) than the ones used experimentally. According to Vöröslakosi et al.,^[^ ^92^ ^]^ in humans, scalp‐applied currents are halved by the skin and subcutaneous soft tissue and further attenuated by the skull by 16%. Additionally, there is an inherent complexity in the distribution and orientation of the induced current due to the cortical folding and the distortion the currents undergo before reaching the cortex. However, we hope that the results presented here could represent a step forward in the development of new or improved brain stimulation strategies for the treatment of disorders associated with pathological oscillations.

## 4 Conclusion

This study investigates the experimental and computational interaction between exogenous electric fields and endogenous cortical network dynamics. By systematically varying parameters of electric fields, we found that changes in frequency and amplitude of stimulation resulted in highly nonlinear interactions, including both phase‐locked and desynchronized cortical states, each occupying defined regions within the parameter plane. As a result, we propose a novel, robust protocol combining DC and AC electric fields that enhances the range of control over cortical dynamics, facilitating a more reliable induction of synchronization or desynchronization of activity, crucial for treating conditions such as those characterized by pathological synchrony. These results not only deepen our understanding of the mechanisms underlying cortical dynamics and their modulation but also provide a foundation for developing precise, targeted brain stimulation therapies that could improve clinical outcomes in neurological and psychiatric disorders.

## 5 Experimental Section

### Preparation of Ferret Cortical Slices

Coronal cortical slices were prepared following the protocol described by Sanchez‐Vives and McCormick.^[^ ^93^ ^]^ Adult ferrets (4–7 months, either sex, *n* = 13) were deeply anaesthetized with an intramuscular injection of 8 mgkg^−1^ of ketamine and 0.1 mgkg^−1^ of medetomidine and decapitated. The entire forebrain was rapidly removed to the oxygenated cold (4–10 °C) bathing medium, and 400‐µm‐thick coronal slices were obtained from the occipital cortex containing primary and secondary visual cortical areas (areas 17, 18, and 19). To increase tissue viability during slice preparation, an adapted sucrose‐substitution technique developed by Aghajanian and Rasmussen was implemented.^[^ ^94^ ^]^ Slices were placed in an interface‐style recording chamber (Scientific Systems Design Inc., Milton, Canada) and bathed for 30 min in an equal mixture of the sucrose‐substituted solution and artificial cerebrospinal fluid (ACSF). Slices were then maintained for 1 h in ACSF for recovery and in an in vivo‐like modified ACSF^[^ ^95^ ^]^ throughout the rest of the experiment. ACSF contains (in mM): NaCl, 126; KCl, 2.5; MgSO_4_, 2; NaH_2_PO_4_, 1; CaCl_2_, 2; NaHCO_3_, 26; dextrose, 10. The in vivo‐like modified ACSF has the same ionic composition, except for different levels of (in mM): KCl, 4; MgSO_4_, 1; and CaCl_2_, 1. Solutions were aerated with 95% O_2_, 5% CO_2_ to a final pH of 7.4. Temperature was kept at 34.5–36°C. Electrophysiological recordings started following a recovery period of at least 40 min after changing to the in vivo‐like modified ACSF. All experiments were conducted in accordance with the European Union Directive 2010/63/EU and approved by the CEEA ethics committee at the University of Barcelona.

### Electrophysiological Recording of Cortical Slices In vitro

Extracellular local field potentials (LFP) were recorded using a 32‐channel multi‐electrode array (MEA). The signal was acquired, amplified, and digitized by a ME2100‐System in combination with the Multi Channel Experimenter software (MCS, Reutlingen, Germany), at a sampling frequency of 5 kHz.

### Cortical Modulation Through Electrical Stimulation

To create a homogenous electric field perpendicular to the cortical layers, two Ag‐AgCl electrodes were placed 6 mm apart parallel to the cortical surface (Figure 1a), similar to previous studies.^[^ ^28^, ^33^, ^56^, ^96^ ^]^ The electrical stimulation protocols were defined in Spike2 (Cambridge Electronic Design, Cambridge, UK), delivered through the Power1401 ADC/DAC (Cambridge Electronic Design) and converted to current through an NL512 Biphasic Buffer combined with two NL800A Stimulus Isolators (Digitimer, Welwyn Garden City, UK). Positive fields are defined as the ones oriented inward, from the cortical surface to the white matter, inducing depolarization of pyramidal neurons, whereas negative fields are oriented in the opposite direction, prompting cellular hyperpolarization (Figure 1a). The intensity of the mean exogenous EFs generated by a given current was calculated as described by Kabakov et al.^[^ ^97^ ^]^ using graphene microtransistor arrays, which were able to provide a direct measure of voltage changes.^[^ ^98^ ^]^ The EF to slices expressing spontaneous were applied SO (Figure 1b). AC fields were delivered for periods of 60 s, with intensities ranging from ±1 to ±8 Vm^−1^ and frequencies varying from 0.05  to 10 Hz. AC fields were also applied with a DC offset of 0 , −2 , or 2 Vm^−1^.

### Computational Model

For a detailed mechanistic understanding of cellular and network AC‐intrinsic rhythm interaction, a model of the cortical network composed of excitatory and inhibitory spiking neurons capable of reproducing the spontaneous activity of cortical slices, such as the one described by Mattia and Sanchez‐Vives^[^ ^48^ ^]^ (Figure 1c), was used. Following Torao‐Angosto et al.,^[^ ^47^ ^]^ the spiking neuron network model consisted of 6880 excitatory and 2580 inhibitory leaky integrate‐and‐fire neurons, each receiving on average 2910 (746) synaptic contacts from excitatory (inhibitory) presynaptic sources. Only 2% of excitatory connections were recurrent. Synaptic efficacies were set to have a fixed point with firing rates of 0.75 Hz (4375 Hz) for excitatory (inhibitory) neurons, respectively. Each neuron had an emission threshold of 20 mV and a reset potential of 15 mV. The refractory period was 2 and 1 ms for excitatory and inhibitory neurons, and the membrane time constants were 20  and 10 ms, respectively. Excitatory (inhibitory) neurons received Poisson background noise with frequencies of 2296 Hz (586.4 Hz). The synaptic transmission was instantaneous, including exponentially distributed axonal delays with means of 22.6 ms (5.7 ms) for presynaptic excitatory (inhibitory) senders. Excitatory neurons received an additional adaptation current with strength *g_a_* and decay time constant τ_*a*_ = 150 ms (Figure 1d).

Stimulations due to an exogenous electric field affect the emission threshold of pyramidal neurons.^[^ ^70^, ^96^ ^]^ In our model, t, his effect was equivalently implemented as an additional current proportional to the field intensity injected into all excitatory neurons.^[^ ^49^ ^]^ A balance was kept between the strength *g_a_* of spike frequency adaptation and the rate of the Poisson background excitatory noise. The network simulations were performed by a custom event‐driven simulator.^[^ ^99^ ^]^ In Figure 8, the same network is simulated by resorting to the integration of the equivalent Fokker‐Planck equation,^[^ ^100^ ^]^ that describes the population dynamics in the mean‐field limit; that is, when the number of neurons is large. This allowed us to access the importance of finite‐size effects in the response of the network to external stimuli spanning a wide section of the (excitation, adaptation) plane.

### Data Analysis

The multi‐unit activity (MUA) was extracted from the LFP signal, high‐pass filtered at 200 Hz. Given the high fluctuations in the firing of the neurons in close proximity to the electrode, the MUA signal values were logarithmically scaled, resulting in a normalized firing rate of the population.^[^ ^99^ ^]^ Then, the Hilbert transform (H) was employed on both the logMUA response signal *s*(*t*) and the sinusoidal perturbation *p*(*t*), yielding their respective analytic signals:

(1) $$s^{a}\left(t\right)=s\left(t\right)+iH\left\{s\left(t\right)\right\}$$

(2) $$p^{a}\left(t\right)=p\left(t\right)+iH\left\{p\left(t\right)\right\}$$

where H{x(t)} is the Hilbert transform of *x*(*t*). From these analytic signals, the instantaneous phase was extracted at each time point *n*, generating phase angles ϕ_*s*_(*n*) and ϕ_*p*_(*n*) for the response and perturbation, respectively.

The phase difference Δϕ_*n*_ between *s^a^* (*t*) and *p^a^* (*t*) at the time point *n* was calculated as:

(3) $$\Delta \;\phi_{n}=\phi_{p}\;\left(n\right)-\phi_{s}\left(n\right)$$

Using this information, histograms in polar coordinates (polar plots) were constructed to visually demonstrate the system's tendency toward a preferred phase difference as the parameter plane (*A*, ν) was traversed, where *A* represents the amplitude of the sinusoidal perturbation and ν its frequency.

The Phase Locking Value (PLV) was a measure commonly employed to quantify the synchronization or phase locking between two oscillatory signals.^[^ ^101^ ^]^ In this study, a modified PLV, termed Amplitude and Phase Locking value (APLV), was introduced to account for amplitude fluctuations that might be unrelated to phase synchronization:

(4) $$APLV=\left|\frac{1}{N}\sum_{n=1}^{N}\frac{e^{i\Delta \phi_{n}}\cdot \left|s_{n}^{a}\right|}{\bar{\left|s^{a}\right|}}\right|$$

where $\bar{s^{a}}$ is the mean of *s^a^* (*t*). APLV values were interpolated along the different ν values using a cubic spline interpolation approach. By employing this method, a smooth curve that passed through all data points, enabling us to analyze the trends and fluctuations in amplitude and phase locking across various stimulation frequencies with enhanced clarity was created.

The Power Spectral Density (PSD) was calculated utilizing the Welch method.^[^ ^102^ ^]^ To do so, the signal was divided into overlapping segments, the Fourier transform was computed for each segment, and averaged the results.

## Conflict of Interest

The authors declare no conflict of interest.

## Data Availability

The data that support the findings of this study are available from the corresponding author upon reasonable request.
